# A comparative evaluation of the bonding efficacy of two-step vs all-in-one bonding agents – An *in-vitro* study

**DOI:** 10.4103/0972-0707.57632

**Published:** 2009

**Authors:** Viresh Chopra, Himanshu Sharma, S Datta Prasad

**Affiliations:** Department of Conservative Dentistry and Endodontics, Subharti Dental College, Meerut, India

**Keywords:** Multibottle system, UniFil Bond, single bottle system, iBond, tensile bond strength

## Abstract

**Aim:**

Aim of this *in vitro* study was to compare the tensile bond strength of UniFil Bond (GC America) vs iBond (Heraeus Kulzer) in conjunction with light cure composite resin (Venus, Heraeus Kulzer).

**Materials and Methods:**

Sixty mandibular molars were taken and divided into 3 groups which were treated with UniFil Bond, iBond and no adhesive respectively. The tensile test was performed using an Instron machine.

**Results:**

The results showed that multibottle systems (UniFil Bond, i.e., two-step)performed 30% better as compared with single bottle systems (all-in-one, i.e., one-step bonding agents).

**Conclusion:**

It can be concluded that UniFil Bond (Multibottle system – 6^th^generation type I) performed better than iBond (Single Bottle system – 7th generation.

## INTRODUCTION

The foundation of modern adhesive dentistry was laid in 1955 when Buonocore reported that acids could be used to alter the surface of enamel to render it more receptive to adhesion.[1]

One of the problems faced in adhesive dentistry is resin-dentin bond degradation by water and vapor over a long period of time. Bonding to enamel remains both the simplest and most reliable of all adhesive procedures.[2] Bonding to dentin is where the clinician faces difficulty. Dentin is about 45% inorganic and the rest being organic and water. Lower bond strength to dentin occur as a result of numerous factors.[4]

1. Dentin contains less mineralized tooth structure and more water than enamel.
2. The presence of smear layer makes the wetting of the dentin very difficult.
3. Fluid-filled channels in dentin that are under slight, but constant, outward pressure from the pulp reduce the stability of the bond between composite resin and dentin.

To overcome these problems, dental adhesive systems have evolved through several generations with changes in chemistry, mechanisms, number of bottles, application techniques and clinical effectiveness.

The present study is performed to assess the tensile bond strength to dentin of two recently introduced dentin bonding agents (UniFil Bond and iBond) used in conjunction with light cure composite (Venus).

## MATERIALS AND METHODS

The study was conducted in the Department of Conservative Dentistry and Endodontics in Subharti Dental College, Meerut, in association with the Department of Mechanical Engineering, IIT, Delhi. The materials and instruments used in this study were as follows:

### Materials

1. UniFil Bond: GC America
2. iBond: Heraeus Kulzer
3. Composite Resin: Venus (Heraeus Kulzer)
4. Etchant: 37% Scotch Bond Multi-Purpose (3M ESPE).

### Methods

Sixty freshly extracted teeth (Mandibular molars) free from all defects were selected. They were stored in normal saline until used for the experiment. The roots of the teeth were embedded in an acrylic mould of a circular shape having a diameter of 12 mm. The occlusal surface of each tooth was reduced with high speed (#245 carbide bur) under constant water spray in order to expose the flat surface of dentine. The specimens were grinded against the 600-sand grit paper mounted on a wheel to obtain a flat dentine test surface. Each specimen was wrapped in gauze moistened with saline and stored in a closed container at room temperature until the adhesive and restorative materials were applied. The teeth were then randomly divided into three groups of 20 teeth each. The groups for the experiment were as follows:

Group I: Twenty teeth were treated according to manufacturer′s instructions with sixth generation type I self-etch adhesive, i.e., two-step adhesive (UniFil Bond, GC America) for the adhesion of composite resin on its dentin surface.

Group II: Twenty teeth were treated according to manufacturer′s instructions with seventh generation single-step self-etch adhesive (iBond-Heraeus-Kulzer) for the adhesion of composite resin on its dentin surface.

Group III: Twenty teeth were treated without adhesive for the adhesion of composite resin on its dentin surface. The third group was used as a negative control group.

Care was taken while preparing the experimental blocks in both the groups so that the centers of all the three components of the experimental design (the acrylic block, tooth, and composite cylinder) coincided in a straight line. This is to avoid any discrepancy in the tensile bond strength evaluation due to the involvement of shear forces that would be generated and would play a role if the centers are not in a straight line.[8]

A composite cylindrical block of with a length of 4 mm was formed over the prepared tooth surface with the help of plastic pipe and bonding agent. The plastic pipe was removed from the cured composite by splitting it with a Bard Parker blade with minimal force. The specimens were stored in distilled water for 24 h.

The specimens were mounted on the Instron machine with a crosshead speed of 0.5 mm/min to calculate the tensile bond strength. They were mounted in such a way that the centers of the experimenting unit (compromising of the acrylic resin, teeth and composite resin block) and the testing bar were in a straight line.

## RESULTS

**Figure 1:**
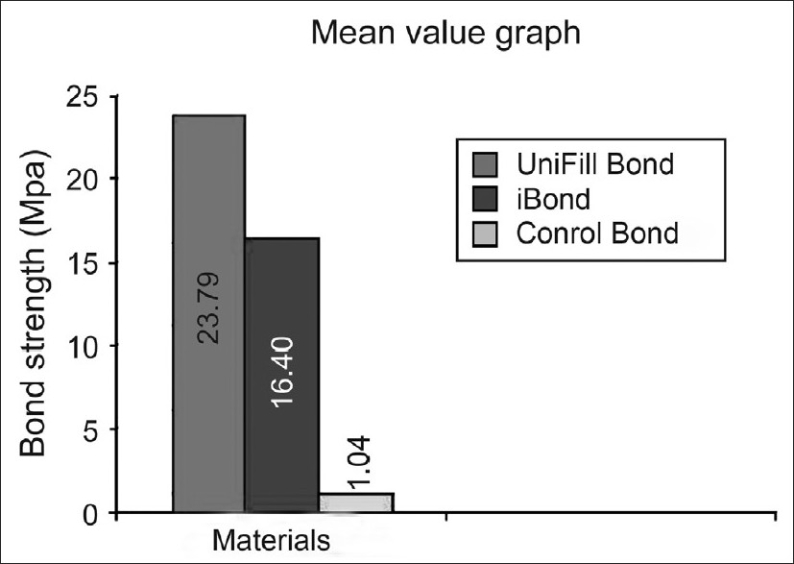
Graphs comparing the maximum, minimum and mean values of all the three groups (I–III)

SPSS and SAS software were used to do the statistical analysis. The comparisons between each group were done by using student ‘t’ statistic and Dunnet's test (an extension of student's ‘t’ test The mean values calculated for each of the group individually are given in Figure 1. The results of this study showed that Group I (UniFil Bond, multibottle system – 6^th^ generation type I) performed better than Group II (iBond, single bottle system – 7^th^ generation).

## DISCUSSION

Bond strength tests are most frequently used to screen test adhesives.[8] The rationale behind this testing method is that the stronger the adhesion between the tooth and the biomaterial, the greater is its resistance to stresses. The importance of high tensile bond strengths in adhesion of resin materials to enamel, particularly to dentin, for the production of well-sealed and long lasting restorations has often been shown in various studies.[7–9] Today's focus in adhesive technology is directed towards simplifying procedures, reduced technique sensitivity and most recently additional chemical interaction with the tooth substance.[9] There is a trend toward eliminating as many steps as possible in the bonding protocol.[10]  This * in vitro * study was performed to verify the performance of latest dentine bonding agents.

The present study compares the tensile bond strength of a multibottle (UniFil Bond, GC America) versus single bottle (iBond, Heraeus Kulzer, Germany) dentine bonding agents.

The superior performance of two-step self-etch systems (Group I) in comparison to one-step self-etch systems (Group II) may be due to the following:

1. Lower pH of UniFil Bond (Group I) in comparison to iBond (Group II).[7]
2. Organic solvents in the form of ethanol are present in UniFil Bond (Group I), whereas acetone is present in iBond (Group II).[7]
3. Low concentration of the solvent present.[11]
4. Low hydrophilicity.[11]
5. Limited etching and demineralization of the underlying dentin over longer period of time.[10]
6. Greater degree of polymerization.[11]

Self-etch adhesives are basically of two types according to pH:[11] Strong adhesives (pH < 1) and mild adhesives (pH > 1).

High acidity for strong self-etch adhesives results in deep demineralization that is equal to phosphoric acid etching.[9] Mild self-etch adhesives dissolve the dentin surface only partially so that a substantial number of hydroxyapatite crystals remain within the hybrid layer. The pH of UniFil Bond used in this study is 2.2; hence, it falls in the category of mild self-etching adhesive.[9] The pH of iBond used in this study is 2, which can be considered to be more acidic than UniFil Bond.

UniFil Bond is an ethanol-based adhesive, and iBond is an acetone-based adhesive.[12]

Ethanol and acetone are organic solvents that act as carriers and water chasers delivering the functional monomers into the hybrid layer.[13] Since the vapor pressure (at 25°C) for acetone is 200 mmHg as compared with 54.1 mmHg for ethanol, acetone is more volatile than ethanol.[13] Kanca showed [14] that the boiling point of acetone is increased and that of water is reduced when acetone-based adhesives are applied to a wet and etched dentin substrate. Both acetone and water then evaporate leaving the resin monomer that envelopes the exposed collagen network. Ethanol works in a similar manner, although it has less capacity for dissolving monomer and a lower vapor pressure than acetone. Another possible explanation to the low bond strength obtained using acetone-based adhesives is the high percentage of acetone (approx 70%), which may not permit the formation of a uniform film on the denting surface.[15]

One-step self-etch adhesives theoretically combine the three functions of the conventional three-step adhesives, i.e., etching, priming and bonding – both hydrophilic and hydrophobic monomers are blended with a relatively high concentration of solvent required to keep them in solution. In this mixture, water is also essential as an ionization medium to enable self-etching activity. This increases the hydrophilic content in iBond in comparison to UniFil Bond, and due to their high hydrophilicity, these adhesives behave as semi-permeable membranes that allow fluids to pass through in comparison to UniFil Bond. This property leads to the degradation of the bond created by one-step self-etch adhesives.[8]

Serious limitations of all-in-one adhesives are as follows: Incomplete polymerization and continued demineralization of the adjacent dentin structure in the tubules.[10]

For all-in-one adhesives to be acidic, the formulations have become more hydrophilic, thereby allowing deeper penetration. As the adhesive penetrates the wet dentinal tubules deeply, the water content increases. Studies have shown that this water acts as a major interfering factor in polymerization which leads to unpolymerized acidic and aggressive monomers to continue etching the dentin, thereby leading to a detrimental impact on the bond.[10] The *in vitro* bond strength of group I (multibottle system, UniFil Bond) and group II (single bottle system-iBond) were higher than the clinically calculated values.[10,11] As the bonding was done on the flat surface the polymerization contraction stress was minimized. Moreover, there was only one bonded surface and three unbounded surfaces, which gave a configuration factor (C-factor) of 0.33, indicating low interfacial stresses.[17] Clinically, the ratio of bonded to unbounded surfaces is high and this increases the C-factor, thereby increasing the interfacial stresses.[17]

Other factors leading to low bond strength values and failure of resin-based adhesives *in vivo* are harsh conditions of the oral environment, such as intraoral temperature, moisture contact, fatigue of bond due to tooth flexure and bacterial enzymes.[14]

On comparing the two experimental groups, it is clear that performance of group I (multibottle system, UniFil Bond) was better than group II (single bottle, iBond) by 30% and provided us with much more durable bonds than the latter adhesive system.

In simpler words, on the basis of this study and various other studies in the literature, two-step self- etch systems showed a superior *in vitro* performance in terms of tensile bond strength in comparison to one-step self-etch systems (all-in-one systems). Thus, the results are in accordance with those of other studies.[11,13,16,18]

## CONCLUSION

It is clear from the present study that tensile bond strength values calculated were higher for multibottle system (UniFil Bond) in comparison to single bottle system (iBond); therefore, the former showed better performance in the *in vitro* study than the latter.

The results of this study show that performance of multibottle systems (UniFil Bond, i.e., two-step) -was better as compared to single bottle system (all-in-one, i.e., one-step bonding agents) by 30%. Thus, it can be concluded that UniFil Bond (Multibottle system – 6^th^ generation type I) performed better than iBond (Single bottle system - 7^th^ generation); however, these results have to be substantiated with further *in vitro* and long-term clinical studies.
